# Is periodontitis a risk factor for ischaemic stroke, coronary artery disease and subclinical atherosclerosis? A Mendelian randomization study

**DOI:** 10.1016/j.atherosclerosis.2020.09.029

**Published:** 2020-11

**Authors:** Steven Bell, Joel T. Gibson, Eric L. Harshfield, Hugh S. Markus

**Affiliations:** Stroke Research Group, Department of Clinical Neurosciences, University of Cambridge, Cambridge, UK

**Keywords:** Periodontitis, Stroke, Coronary artery disease, Carotid intima-media thickness, Mendelian randomization, Inflammation, Risk factor

## Abstract

**Background and aims:**

Observational studies have reported an association between periodontitis and cardiovascular disease but whether this association is causal is uncertain. We therefore used Mendelian randomization to test whether periodontitis is causally associated with stroke, coronary artery disease, or subclinical atherosclerosis.

**Methods:**

A two-sample Mendelian randomization analysis was carried out using five single nucleotide polymorphisms previously associated with periodontitis in genome-wide association studies. Summary data were drawn from MEGASTROKE and combined with *de novo* analyses of UK Biobank for stroke and its major subtypes (up to 44,221 cases, 739,957 controls) and CARDIoGRAMplusC4D and UK Biobank for coronary artery disease (122,733 cases, 424,528 controls). We used existing data on carotid intima-media thickness in UK Biobank as a marker of subclinical atherosclerosis (N = 22,179). Causal estimates were obtained using inverse-variance weighted Mendelian randomization. Sensitivity analyses were performed using weighted median and MR-Egger approaches.

**Results:**

No association was found between periodontitis and any stroke (odds ratio [OR] per doubling in the odds of periodontitis 0.99, 95% confidence interval [CI] 0.97 to 1.02), ischaemic stroke (OR 1.00, 95% CI 0.97 to 1.03) or its major subtypes (*p* > 0.4), or coronary artery disease (OR 1.01, 95% CI 0.99 to 1.03). Similarly, we found no association for periodontitis and subclinical atherosclerosis (β −0.002, 95% CI -0.004 to 0.001). These results were consistent across a series of sensitivity analyses.

**Conclusions:**

These findings provide no robust evidence for a causal relationship between periodontitis and stroke or coronary artery disease. This suggests that associations reported in observational studies may represent confounding.

## Highlights

• Periodontitis and cardiovascular disease correlate in observational studies.
• Mendelian randomization does not support a causal association.
• Results do not support treatment of periodontitis to reduce atherosclerotic disease.

## Graphical abstract

**Figure:**
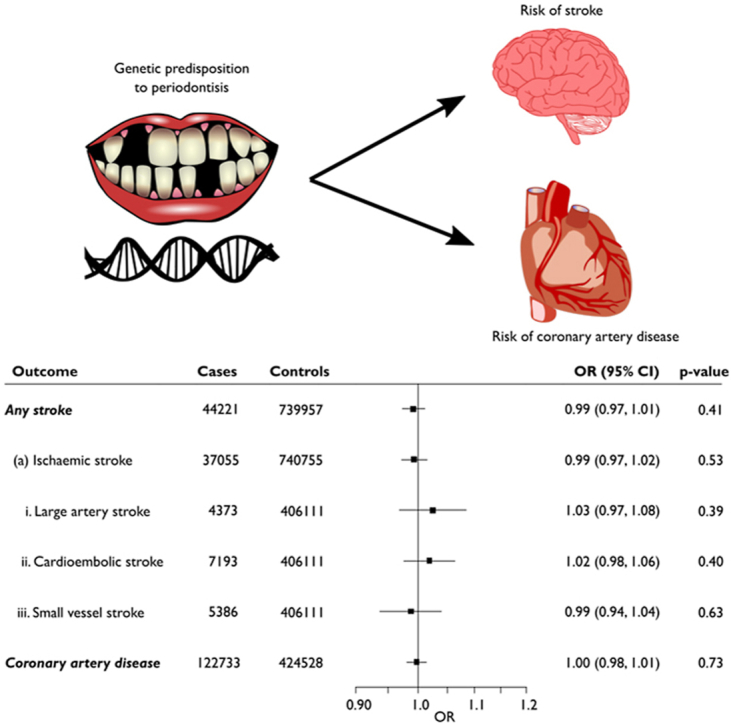

## 1 Introduction

Cardiovascular disease is the leading cause of mortality worldwide. Despite improved treatment options and disease prevention strategies, cardiovascular disease continues to be responsible for around 17.8 million deaths each year [1]. Traditional risk factors include age, hypertension and dyslipidaemia [2], but the role of inflammation in the pathogenesis and progression of the disease is increasingly recognised [3].

Periodontitis is a chronic inflammatory condition affecting the supporting tissues surrounding the teeth [4]. This disease is triggered by an immune response to oral bacteria, and has been associated with cardiovascular disease [5]. Estimates of the prevalence of periodontitis are high, ranging from 20 to 50% of the general population [6], so determining whether the reported association is causal of cardiovascular disease is an important issue for public health.

It has been hypothesised that periodontitis increases the risk of cardiovascular disease through systemic inflammation and an immune-mediated response [7]. The disease starts locally within the periodontium where oral bacteria accumulate and infiltrate the tissues surrounding the teeth. This causes local inflammation and an immune response, leading to the destruction of the surrounding tissues and typical clinical features on dental examination [4]. This damage increases the risk of pathogen entry into the bloodstream, where they may spread causing systemic inflammation and disease [8]. An increase in systemic inflammatory biomarkers, such as interleukin-6, C-reactive protein and tumour necrosis factor-alpha, has been observed in both periodontitis and cardiovascular disease [9,10].

Several mechanisms by which periodontitis leads to atherosclerosis have been proposed. The release of toxic cysteine proteases (gingipains) by oral bacteria is thought to irritate endothelial cells [11] and promote atherosclerosis through endothelial cell inflammation and lipid deposition [12]. Alternatively, the release of proinflammatory cytokines and chemokines may cause a downstream immune response, where the resultant antibodies react with endothelial cells and low-density lipoproteins, promoting the atherosclerotic changes [7].

Observational studies have associated periodontitis with a range of diseases, including cardiovascular disease [5], Alzheimer's disease [13], hypertension [14], and diabetes [15]. A recent meta-analysis found that individuals with periodontitis had almost two-fold greater risk of stroke, with this effect further pronounced for events of ischaemic origin [16]. However, observational studies are subject to confounding; for example, those with periodontal disease may have poorer access to healthcare or lower income [17]. Therefore, evidence of a causal link between periodontitis and stroke and coronary artery disease is lacking.

Mendelian randomization (MR) is a technique used for assessing causal relationships between risk factors and disease outcomes [18]. MR uses genetic variants associated with a risk factor as instrumental variables (IVs), which can be tested for associations with disease outcomes. For robust causal inferences to be made, all IVs used must satisfy three assumptions. For an IV to be valid it must be associated with the exposure of interest, it must only affect the outcome via this exposure, and it must not be associated with confounders of the exposure-outcome relationship [19]. In this way, if variants associated with a risk factor are also associated with the disease, then this strengthens the evidence of a causal effect of the risk factor on the disease.

In this study, we examined data from genome-wide association studies (GWAS) of clinically-defined periodontitis, stroke, coronary heart disease, and carotid intima-media thickness (cIMT) as a marker of subclinical atherosclerosis to determine whether periodontitis is causally associated with these common cardiovascular disease subtypes.

## 2 Materials and methods

### 2.1 Study overview

This study used MR as a method of evaluating whether a causal relationship exists between periodontitis and stroke, ischaemic stroke and its major subtypes, coronary artery disease, and subclinical atherosclerosis proxied by cIMT. This was undertaken using a two-sample MR approach using summary data, where the SNP-exposure (periodontitis) and the SNP-outcome (stroke, coronary artery disease and cIMT) associations were taken from different sources [20].

### 2.2 Selection of instrumental variables

All SNPs used in this study were identified from recent GWAS of clinically-confirmed periodontitis in German, Dutch or European American samples or from meta-analyses of these studies [[21], [22], [23]]. Candidate SNPs were assessed for suitability against the assumptions required of a valid IV described above. Only SNPs associated with periodontitis at a genome-wide significance level (*p* < 5 × 10^−8^) were included, and findings were required to be replicated in an external study. Where multiple SNPs were identified at the same locus, only the "lead" SNP (i.e., with the smallest *p*-value) was included. Studies relying on self-reported phenotypes were excluded, as were studies reporting only rare variants. A total of five SNPs were identified for inclusion in our analysis (Table 1).

**Table 1:** Single nucleotide polymorphisms associated with periodontitis.

rsID	Locus	Nearest gene	EA	NEA	EAF	OR (95% CI)	*p*-value	PVE (%)	Reference
rs1537415	9q34.3	*GLT6D1*	C	G	0.41	1.59 (1.36–1.86)	5.51 × 10^−9^	1.88	[21]
rs4284742	19q13.41	*SIGLEC5*	G	A	0.76	1.34 (1.21–1.48)	1.34 × 10^−8^	0.31	[22]
rs2738058	8p23.1	*DEFA1A3*	T	C	0.43	1.28 (1.18–1.38)	6.78 × 10^−10^	0.35	[22]
rs16870060	8q22.3	*MTND1P5*	G	T	0.91	1.36 (1.23–1.51)	3.69 × 10^−9^	0.28	[23]
rs729876	16p13.12	*LOC107984137*	T	C	0.82	1.24 (1.15–1.34)	9.77 × 10^−9^	0.25	[23]

EA, effect allele; NEA, non-effect allele; EAF, effect allele frequency; OR (95% CI), odds ratio (95% confidence interval); PVE, proportion of variance explained.

Alleles were orientated so that the effect allele was the allele which increased risk of periodontitis. Effect sizes were taken as the natural-log of the odds ratio (OR) per periodontitis risk-increasing allele. One palindromic SNP (rs1537415) was harmonised between studies using the minor allele frequency reported for each population. Here we report the effect allele as being the minor C allele. Summary data was used to calculate the proportion of variance in periodontitis explained by each SNP [24]. From this we calculated an expected F statistic using the formula $F=\frac{N-K-1}{K}\times \frac{R^{2}}{1-R^{2}}$ , where *N* is the sample size of the exposure dataset, *K* is the number of genetic variants used as instrumental variables, and R^2^ if the proportion of variance explained in the exposure dataset by these variants [25]. As our instruments were drawn from three discovery studies we used the mean sample size (calculated as 8254) of these to define *N* and relatedly, because the proportion of variance explained in the exposure by the set of five variants was not reported in any of the original investigations we selected the highest proportion of variance explained by a single SNP (1.9%; Table 1) for the value of R^2^ (this is likely to be overly conservative). Our expected F statistic was therefore calculated as $\frac{8248}{5}\times \frac{0.019}{1-0.019}$ = 32.0 with the lower limit of the one-sided 95% confidence interval (95% CI) of this being 24.1 indicating considerable weak instrument bias would not be expected. As these SNPs were detected in predominantly European cohorts, all further analyses for the stroke outcomes were restricted to European populations.

Each SNP was searched in PhenoScanner V2 [26,27] to check for signs of pleiotropy. This search was restricted to identify any diseases and traits associated with the five SNPs at a genome-wide significance level (*p* < 5 × 10^−8^) and included all proxy SNPs (r^2^ > 0.8 in 1000 Genomes phase 3). The search output was limited to Europeans only. One of these SNPs (rs2738058) was associated with immune cell counts [28] but was determined to likely reflect vertical, not horizontal, pleiotropy, so was not excluded. No other SNP was associated with any other phenotype.

### 2.3 Outcome study populations

**Fig. 1:**
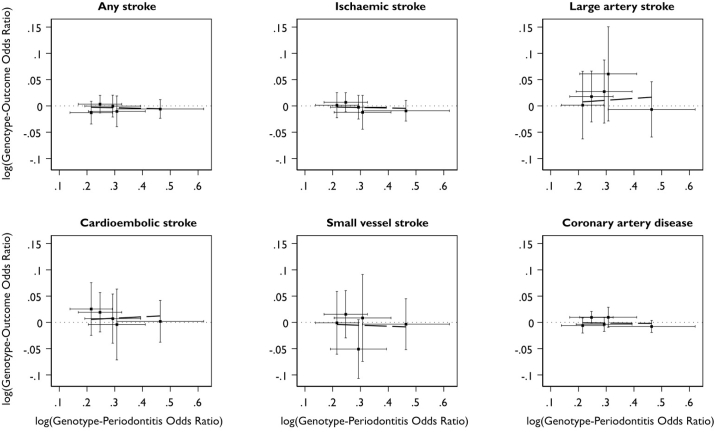
Cross-hair plots of genotype-periodontitis and genotype-outcome associations for stroke and coronary artery disease.

Estimates of the SNP-outcome associations for all stroke, ischaemic stroke and its major subtypes were drawn from the MEGASTROKE consortium European ancestry cohort [29]. To increase the power of our analyses, data for the association of the five periodontitis variants with any stroke and ischaemic stroke were combined with association estimates derived from our own analysis of UK Biobank (using algorithmically defined events released by the study [30] which are a combination of participant self-reported data and linked electronic health records covering primary and secondary care as well as mortality registries [ICD9 codes 434.X and 436.X for ischaemic stroke as well as 430.X and 431.X for any stroke; ICD10 codes I63.X and I64.X for ischaemic stroke plus I60.X and I61.X for any stroke]) using a fixed effects inverse-variance weighted (IVW) meta-analysis. This was carried out using the metafor package in R (v3.6.2) [31]. Estimates from the UK Biobank data were calculated using logistic regression assuming an additive genetic model, adjusting for age, sex and 10 principal components of ancestry in SNPTEST v2.5.2 [32]. The combined cohort included data from 44,221 stroke cases and 739,957 controls. The number of cases and controls used for each stroke subtype is reported in Fig. 1. SNP association data for coronary artery disease were taken from a recent genome-wide meta-analysis of the CARDIoGRAMplusC4D and UK Biobank datasets [33]. This cohort comprised 122,733 cases and 424,528 controls. Data for the association of SNPs with subclinical atherosclerosis were drawn from the latest and single largest study with consistent cIMT measures in 22,179 UK Biobank participants [34].

### 2.4 Mendelian randomization

Estimates derived from an IVW MR analysis were used as the primary outcome measures of this study [35]. This used a random-effects model with simple weights based on the first-order term of the delta expansion for the variance.

### 2.5 Sensitivity analysis

Sensitivity analyses were performed using the weighted median and MR-Egger methods since IVW MR only produces reliable results when all SNPs satisfy the IV assumptions. These alternative MR approaches relax some of these strict requirements so they can produce robust results even when some IVs are invalid. The weighted median method allows for invalid IVs to be included and provides consistent estimates if at least 50% of the weight of the analysis comes from valid IVs. The MR-Egger method allows for each IV to exhibit pleiotropy and is consistent if the instrument strengths are independent of these pleiotropic effects (InSIDE assumption). The MR-Egger method additionally provides a means of testing for directional pleiotropy. For all IVW and MR-Egger models, studentised residuals were calculated to identify any outlying IVs. Overly influential IVs were identified using Cook's distance. An IV was considered an outlier if the magnitude of its studentised residual was greater than 3. An IV was considered highly influential if its Cook's distance was greater than 4/n, where n was the number of variants included in the analysis. We further supplemented these analyses with Mendelian Randomization Pleiotropy RESidual Sum and Outlier (MR-PRESSO) global tests [36].

All MR analyses, other than MR-PRESSO, were performed using the Mendelian Randomization package [37] in R and results rescaled to represent per doubling of the odds of periodontitis through multiplying the log causal estimate (and associated 95% CI) by 0.693 and then exponentiating [38]. Forest plots were produced using the forestplot package [39]. A *p*-value < 0.05 was considered significant and *post hoc* power calculations were performed (Supplementary Table 1) to determine whether we had adequate statistical power to detect positive causal effects should they exist.

### 2.6 Genetic correlations

In a *post hoc* analysis, we also assessed the shared genetic liability of periodontitis with stroke, coronary artery disease and subclinical atherosclerosis using LD Score regression [40,41]. We did so to complement our Mendelian randomization analyses that focussed on ascertaining whether a causal association exists, with estimates of SNP-based coheritability which may reveal whether genetic correlation (i.e., inclusive of horizontal pleiotropy which may reflect shared biological mechanisms) is a reason for why periodontitis and cardiovascular outcomes co-occur frequently in the population. Here we used pre-calculated linkage disequilibrium weights for European ancestry populations and extracted exposure and outcome data from the latest GWAS of periodontitis [42] and the cardiovascular studies reported above (MEGASTROKE without new analyses in UK Biobank) restricted to HapMap 3 SNPs as recommended by the LD Score regression developers.

## 3 Results

### 3.1 Proportion of phenotypic variance explained by each SNP

The proportion of variance in periodontitis explained by each SNP was low (Table 1). The highest proportion was seen for rs1537415, which explained 1.9% of the variance in periodontitis seen in that study. The other SNPs each explained approximately 0.3% of the phenotypic variance observed in their respective cohorts.

### 3.2 Association of periodontitis with stroke and coronary artery disease

**Fig. 2:**
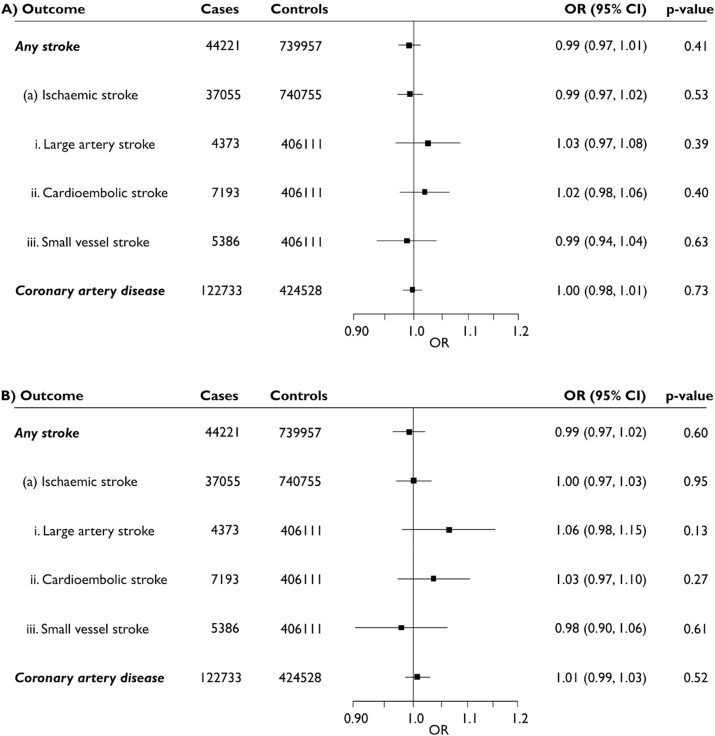
Causal association estimates for a doubling in the odds of periodontitis with risk of stroke and coronary artery disease obtained from inverse-variance weighted Mendelian randomization. Estimates are shown from analyses using (A) all 5 SNPs and (B) following exclusion of rs1537415. OR (95% CI), odds ratio (95% confidence interval).

Cross-hair plots of genotype-periodontitis and genotype-outcome associations are presented in Fig. 1. Estimates obtained from the IVW MR did not reveal an association between periodontitis and stroke or any its subtypes, or coronary artery disease (Fig. 2A). No significant findings were identified from either of the weighted median or MR-Egger sensitivity analyses either. Analysis with the MR-Egger method did not detect any significant directional pleiotropy in any of the outcomes.

### 3.3 Association of periodontitis with subclinical atherosclerosis

We similarly found no association between a doubling in the odds of periodontitis and cIMT (β −0.002, 95% CI -0.005 to 0.001) in our primary or sensitivity analyses.

### 3.4 Removal of potentially outlying SNPs

Calculation of studentised residuals for each linear MR model did not reveal any consistently outlying SNPs (Table 2). However, Cook's distance identified rs1537415 to be a highly influential SNP in all but two of the MR analyses. To investigate whether rs1537415 biased our findings, analyses were repeated excluding this SNP and this made little difference to our overall conclusions (Fig. 2B). This is consistent with our MR-PRESSO global test results for each trait (*p* > 0.5 for all stroke outcomes, 0.12 for coronary artery disease and 0.37 for cIMT).

**Table 2:** Studentised residuals and Cook's distances calculated for stroke and coronary artery disease models.

Outcome	Coronary artery disease					Any stroke					Ischaemic stroke				

Model	IVW		MR-Egger		MR-PRESSO	IVW		MR-Egger		MR-PRESSO	IVW		MR-Egger		MR-PRESSO

*rsID*	*SR*	*CD*	*SR*	*CD*	*p*_*Global*_	*SR*	*CD*	*SR*	*CD*	*p*_*Global*_	*SR*	*CD*	*SR*	*CD*	*p*_*Global*_
rs1537415	−1.24	1.55	−0.56	2.88	0.12	−0.08	0.01	−0.39	1.44	0.83	−1.11	1.25	0.80	4.93	0.77
rs4284742	−0.28	0.02	−0.50	0.04		0.44	0.04	0.39	0.03		0.06	<0.01	−0.28	0.01	
rs2738058	2.32	0.48	1.50	0.56		1.37	0.31	1.80	0.69		2.94	0.61	1.88	0.72	
rs16870060	0.92	0.08	0.78	0.04		−0.66	0.05	−0.53	0.02		−0.89	0.08	−1.72	0.09	
rs729876	−0.51	0.03	−1.70	0.48		−1.93	0.18	−2.33	0.60		0.42	0.02	−0.40	0.06	
Outcome	Large artery stroke					Cardioembolic stroke					Small vessel stroke				

Model	IVW		MR-Egger		MR-PRESSO	IVW		MR-Egger		MR-PRESSO	IVW		MR-Egger		MR-PRESSO

*rsID*	*SR*	*CD*	*SR*	*CD*	*p*_*Global*_	*SR*	*CD*	*SR*	*CD*	*p*_*Global*_	*SR*	*CD*	*SR*	*CD*	*p*_*Global*_
rs1537415	−2.34	2.76	−3.42	17.59	0.50	−1.37	1.72	4.32	19.31	0.79	0.28	0.11	0.85	5.59	0.53
rs4284742	0.73	0.11	0.48	0.04		−0.04	<0.01	−0.71	0.07		−4.47	0.64	−5.34	0.35	
rs2738058	0.46	0.06	−0.11	0.01		1.33	0.30	0.42	0.10		1.04	0.22	1.05	0.41	
rs16870060	1.85	0.18	2.01	0.10		−0.56	0.03	−1.86	0.10		0.33	0.01	0.27	<0.01	
rs729876	−0.22	0.01	−1.09	0.29		1.58	0.14	0.80	0.19		0.10	<0.01	0.06	<0.01	

IVW, inverse-variance weighted; SR, studentised residual; CD, Cook's distance; *p*_Global_, *p*-value from MR-PRESSO global test.

### 3.5 Genetic correlation between periodontitis and cardiovascular outcomes

We observed no significant genetic correlation between periodontitis and any of the phenotypes investigated: Rg_any stroke_ = −0.02 (*p* = 0.92), Rg_ischaemic stroke_ = 0.09 (*p* = 0.70), Rg_large artery stroke_ = 0.65 (*p* = 0.60), Rg_cardioembolic stroke_ = −0.01 (*p* = 0.97), Rg_small vessel stroke_ = −0.09 (*p* = 0.83), Rg_coronary artery disease_ = 0.10 (*p* = 0.43), and Rg_cIMT_ = 0.52 (*p* = 0.27).

## 4 Discussion

This study examined whether periodontitis was causally associated with subclinical atherosclerosis, coronary artery disease or stroke using two-sample MR. Despite observational epidemiological data suggesting a two-fold increase in cardiovascular disease risk in patients with periodontitis, this study found no reliable evidence of a causal association between the two.

Only five independent SNPs associated with clinically defined periodontitis at a genome-wide significance level were identified for inclusion in this study. This is somewhat small considering the number of GWAS which have been carried out on the disease. Furthermore, these five SNPs were all identified in cohorts comprising only aggressive periodontitis patients, or a mix of both aggressive and chronic periodontitis patients. Despite chronic periodontitis being the predominant form of the disease, GWAS using solely chronic periodontitis cohorts have failed to identify any genome-wide significant variants associated with the disease [[43], [44], [45], [46], [47], [48]]. To further examine whether chronic periodontitis might be a risk factor for cardiovascular disease, we also examined the shared genetic contribution of chronic periodontal disease and all cardiovascular traits considered in this study, but found no significant evidence of genetic overlap. This also argues against shared biological mechanisms underlying periodontitis and cardiovascular disease.

Confounding may offer an explanation as to why a causal association was not found in this study, despite observational studies suggesting otherwise. Periodontitis and cardiovascular disease are both multifactorial diseases, manifesting in individuals following many years of exposure to risk factors. Therefore, it is possible that the observed association between the two diseases is confounded by a range of other influences [49]. For example, smoking is an important risk factor shared by both periodontitis and cardiovascular disease [50,51]. Other possible confounders include diabetes and socioeconomic position. Diabetes in particular is a known contributor to cardiovascular disease risk, and there is now evidence supporting its role in the development of periodontitis [15]. However, taken together our data does not support a causal relationship between periodontitis and cardiovascular disease, and therefore provides little rationale for prioritising treatment of periodontitis to reduce stroke and coronary artery disease risk.

A previous MR study has reported an association between periodontitis and blood pressure, although the association accounted for only a very small portion of blood pressure variation [52]. We replicated this association in our study (see Supplementary Table 2), but still found no robust association between periodontal SNPs and stroke, coronary artery disease or subclinical atherosclerosis. Hypertension is a well-established risk factor for cardiovascular disease, but it is likely that the contribution of periodontitis to hypertension is so small that a change in cardiovascular risk is not detectable.

Our study had several strengths. These include the use of multiple sensitivity analyses to support all results obtained. The IVW, weighted median and MR-Egger methods all failed to identify a causal association between periodontitis and the cardiovascular disease outcomes. The concordance of results obtained using these different approaches indicates that our findings were robust. Another strength was that only SNPs associated with clinically-defined periodontitis at a genome-wide significance level, and that were replicated in additional cohorts, were included as IVs. This increased the likelihood that all SNPs used were truly associated with periodontitis. Further strengths were the consistency of findings across both coronary artery disease and stroke, and the large sample sizes (and therefore adequate statistical power to detect even relatively modest causal effects; see Supplementary Table 1) used for both outcomes, as well as also observing a null association with subclinical atherosclerosis as proxied by cIMT.

The study also had limitations. The variance explained by the five periodontal disease SNPs was relatively low (although our conservatively calculated expected F statistic did not raise concerns of weak instrument bias) and the selected variants have only been found to associate with aggressive forms of the disease as highlighted above. Furthermore, our genetic instruments were selected using only the lead SNP from each locus, not a list filtered on the basis of a linkage disequilibrium threshold as is often done (n.b., none of our genetic variants were in linkage disequilibrium). It is therefore plausible that more instruments may have been available for our analysis, however, the variants we chose were drawn from three separate GWAS for which full summary data were regrettably unavailable to prune each locus based on linkage disequilibrium. Our results were further supported by the lack of significant evidence of genetic overlap. Genetic correlations for large artery stroke and cIMT were larger than those for other phenotypes, which is interesting given recent observational findings showing that carotid atherosclerosis appears to be an important risk factor only for large artery and lacunar but not cardioembolic stroke [53]. However, these genetic correlations displayed large variance and while it is possible that with the availability of larger, more precise data from future GWAS this may be reduced, it would be premature to conclude that a causal association may exist when considered in tandem with the null findings presented elsewhere in the manuscript. It is also important to consider our findings with the underlying assumptions and general limitations of the Mendelian randomization approach in mind. For example, estimates derived using Mendelian randomization analyses generally reflect the effect of life-long exposure to a risk factor and therefore do not always correspond exactly to the expected outcome of a particular intervention (which may differ in its duration, timing and mechanism targeted) [54,55].

To conclude, this study found no robust evidence of a causal association of periodontitis with stroke, coronary artery disease, or subclinical atherosclerosis. This result contrasts with observational studies, which have suggested an association between these diseases. The null findings reported here indicate this is possibly confounded (by known or unknown sources) and therefore highlight a role for shared risk factors of both diseases.

## Financial support

This work was supported by a 10.13039/501100000274British Heart Foundation Programme Grant (RG/16/4/32218). J.T. Gibson was supported by the 10.13039/501100003343Cambridge Commonwealth, European & International Trust. H.S. Markus is supported by a 10.13039/501100000272National Institute for Health Research (NIHR) Senior Investigator award, and his work is supported by the Cambridge Universities NIHR Comprehensive 10.13039/100014461Biomedical Research Centre. The funding organisations had no role in any of the following: design and conduct of the study; collection, management, analysis, and interpretation of the data; review, or approval of the manuscript.

## CRediT authorship contribution statement

**Steven Bell:** Conceptualization, Methodology, Investigation, Supervision, Visualization, Writing - original draft. **Joel T. Gibson:** Methodology, Investigation, Visualization, Writing - original draft. **Eric L. Harshfield:** Investigation, Writing - review & editing. **Hugh S. Markus:** Investigation, Supervision, Funding acquisition, Writing - original draft.

## Declaration of competing interest

The authors declare that they have no known competing financial interests or personal relationships that could have appeared to influence the work reported in this paper.
