# Supplementary material for: Is periodontitis a risk factor for ischaemic stroke, coronary artery disease and subclinical atherosclerosis? A Mendelian randomization study
Source: Atherosclerosis. 2020 Nov;313:111–7. doi: 10.1016/j.atherosclerosis.2020.09.029 (PMC7660116; doi:10.1016/j.atherosclerosis.2020.09.029)
Supplement: Multimedia component 1 [file mmc1.docx]

*Supplementary material to:*

**Is periodontitis a risk factor for ischaemic stroke, coronary artery disease and subclinical atherosclerosis? A Mendelian randomisation study**

# Power calculations

Post-hoc power calculations were performed using <https://shiny.cnsgenomics.com/mRnd>. We calculated power, using a Type-1 error rate of 0.05, based on achieved sample size in the outcome dataset and used an R^2^ of 0.02 for the variance explained in the exposure.^1^ We varied the “true” odds ratio of the outcome per standard deviation increase in the exposure from 1.0 (null causal effect), 1.05, 1.10, 1.20 and 1.30 (for our only continuous outcome, cIMT, we performed calculations using “true” beta values of 0, 0.05, 0.10, 0.20 and 0.30).

**Supplementary Table 1.** Post-hoc power calculations for Mendelian randomization analyses performed at varying causal effect sizes

|  |  |  | **Power (%)** | | | | |
| --- | --- | --- | --- | --- | --- | --- | --- |
|  | **N** | **K** | **Null causal effect** | **OR=1.05** | **OR=1.1** | **OR=1.2** | **OR=1.3** |
| **Any stroke** | 784178 | 0.06 | 5 | 32 | 84 | 100 | 100 |
| **Ischaemic stroke** | 777810 | 0.05 | 5 | 27 | 77 | 100 | 100 |
| **Large artery stroke** | 410484 | 0.01 | 5 | 7 | 15 | 44 | 77 |
| **Cardioembolic stroke** | 413304 | 0.02 | 5 | 10 | 25 | 72 | 97 |
| **Small vessel stroke** | 411497 | 0.01 | 5 | 7 | 15 | 44 | 77 |
| **Coronary artery disease** | 547261 | 0.22 | 5 | 57 | 99 | 100 | 100 |
| **cIMT****^†^** | 22179 | -- | 5 | 18 | 55 | 98 | 100 |

N, sample size; K, proportion of cases in the study; OR, odds ratio; ^†^ power calculations for cIMT are for beta values of 0, 0.05, 0.1, 0.2 and 0.3, not odds ratios; Type-1 error rate of 0.05; R^2^ for variance explained in the exposure = 0.02

# Replication of blood pressure associations

To assess the suitability of the chosen periodontitis variants for Mendelian randomization (MR) analysis, these SNPs were first used to replicate the results of a recent MR study that found a causal association of periodontitis and hypertension.^2^ The aim of replicating this study was both to verify their results and determine whether the five SNPs used here behave in a similar way. For brevity, only the analysis for systolic blood pressure (SBP) is reported. SNP-SBP association estimates were taken from the latest meta-analysis of UK Biobank and International Consortium of Blood Pressure GWAS data.^3^

**Supplementary Table 2.** Causal association estimates between periodontitis and systolic blood pressure from a series of Mendelian randomisation analyses.

| ***Systolic blood pressure*** | |  |  |  |  |
| --- | --- | --- | --- | --- | --- |
| **Method** | **Estimate** | **SE** | **L95 CI** | **U95 CI** | **p-value** |
| IVW | 0.105 | 0.049 | 0.009 | 0.200 | 0.032 |
| Weighted median | 0.110 | 0.060 | -0.008 | 0.229 | 0.067 |
| Egger | -0.086 | 0.168 | -0.415 | 0.242 | 0.608 |
| Egger Intercept | 0.065 | 0.055 | -0.042 | 0.173 | 0.234 |

IVW, Inverse Weighted; SE, Standard Error; L95 CI; Lower 95% Confidence Interval; U95 CI, Upper 95% Confidence Interval

**References**

1. Burgess S, Davey Smith G, Davies N, Dudbridge F, Gill D, Glymour M, Hartwig F, Holmes M, Minelli C, Relton C, et al. Guidelines for performing Mendelian randomization investigations [version 2; peer review: 2 approved]. *Wellcome Open Res.* [Internet]. 2020;4. Available from: https://wellcomeopenresearch.org/articles/4-186/v1

2. Czesnikiewicz-Guzik M, Osmenda G, Siedlinski M, Nosalski R, Pelka P, Nowakowski D, Wilk G, Mikolajczyk TP, Schramm-Luc A, Furtak A, et al. Causal association between periodontitis and hypertension: evidence from Mendelian randomization and a randomized controlled trial of non-surgical periodontal therapy. *Eur. Heart J.* 2019;40:3459–3470.

3. Evangelou E, Warren HR, Mosen-Ansorena D, Mifsud B, Pazoki R, Gao H, Ntritsos G, Dimou N, Cabrera CP, Karaman I, et al. Genetic analysis of over 1 million people identifies 535 new loci associated with blood pressure traits. *Nat. Genet.* 2018;50:1412–1425.
